# The Risk Factors for Nosocomial Infection in Chinese Patients with Active Rheumatoid Arthritis in Shanghai

**DOI:** 10.5402/2012/215692

**Published:** 2012-03-19

**Authors:** Wei-Lin Xie, Zhuo-Ling Li, Zhen Xu, Huan-Ru Qu, Luan Xue, Xiao Su, Qiang-Hua Wei, Hui Wang, Miao-Ying Li, Fu-Tao Zhao, Lin-Di Jiang, Jiong Zhang, Wei-Guo Wan, Min Dai, Cheng-De Yang, Jian-Long Guan, Li Su, Dong-Bao Zhao, Dong-Yi He, Hu-Ji Xu, He-Jian Zou, Chun-De Bao

**Affiliations:** ^1^Department of Rheumatology and Immunology, Changhai Hospital, Second Military Medical University, Shanghai 200433, China; ^2^Department of Rheumatology and Immunology, Shanghai Guanghua Hospital, Shanghai 200052, China; ^3^Department of Rheumatology and Immunology, Changzheng Hospital, Second Military Medical University, Shanghai 200003, China; ^4^Department of Rheumatology and Immunology, Shanghai Longhua Hospital, Shanghai 200032, China; ^5^Department of Rheumatology and Immunology, Yueyang Hospital of Integrated Traditional and Western Medicine, Shanghai 200473, China; ^6^Department of Rheumatology and Immunology, Shanghai Municipal Hospital of Traditional Chinese Medicine, Shanghai 200071, China; ^7^Department of Rheumatology and Immunology, Shanghai First People's Hospital, Shanghai 201620, China; ^8^Department of Rheumatology and Immunology, Shidong Hospital, Shanghai 200438, China; ^9^Department of Rheumatology and Immunology, No.3 People's Hospital Affiliated to Shanghai Jiao Tong University School of Medicine, Shanghai 201900, China; ^10^Department of Rheumatology and Immunology, Zhongshan Hospital, Fudan University, Shanghai 200032, China; ^11^Department of Rheumatology and Immunology, Huashan Hospital, Shanghai 200040, China; ^12^Department of Rheumatology and Immunology, Renji Hospital, Shanghai 200003, China; ^13^Department of Immunology and Rheumatology, Huadong Hospital, Fudan University, Yananxi Road, Shanghai 200040, China

## Abstract

*Objective*. To analyse the potential risk factors of nosocomial infections in patients with active rheumatoid arthritis (RA). *Methods*. A total of 2452 active RA patients at Hospitals in Shanghai between January 2009 and February 2011 were analyzed. Their demographic and clinical characteristics were compared with those without infection, and the potential risk factors were determined by logistic regression analysis. *Results*. Multivariate analysis indicated the gender (OR = 0.70, 95% CI 0.53–0.92), duration in hospital (OR = 1.03 , 95%CI 1.01–1.05), number of organs involved (OR = 0.82, 95%CI 0.72–0.92), number of disease-modifying antirheumatic drugs ((DMARDs) (OR = 1.22, 95%CI 1.061–1.40)), corticosteroid therapy (OR = 1.02, 95%CI 1.01–1.03), peripheral white blood cell counts ((WBC) (OR = 1.04, 95%CI 1.00–1.08)), levels of serum albumin (OR = 0.98, 95%CI 0.97–0.99), and C-reactive protein ((CRP) (OR = 1.03 , 95%CI 1.01–1.04)) that were significantly associated with the risk of infections. *Conclusion*. The female patients, longer hospital stay, more organs involved, more DMARDs, corticosteroid usage, high counts of WBC, lower serum albumin, and higher serum CRP were independent risk factors of infections in active RA patients.

## 1. Introduction

RA is a chronic inflammatory autoimmune disease with unknown etiology and has an increased risk of infection compared with the general population [1]. Previous studies have shown that microbial infection, particularly for genitourinary and bronchopulmonary infection, contributes to increased rate of mortality in RA patients [2, 3]. It has been a serious concern that RA patients acquire microbial infection during hospitalization. Notably, about 5–10% of RA patients may acquire a microbial infection after their admission, and the hospital-related infection rate in RA patients has been increasing in the USA and other countries during the past decades [4]. Those patients acquire infection with the common hospital-related drug-resistant pathogens, including methicillin-resistant *Staphylococcus aureus* (MRSA), antibiotic-resistant Gram-negative bacilli and, more recently, vancomycin-resistant enterococci [5]. More importantly, these nosocomial infections are difficult to control, leading to a high mortality, particularly in individuals with immunodisorder. Therefore, understanding potential risk factors associated with the high susceptibility will be of great significance in the prevention and control of nosocomial infection.

 RA patients have unbalanced immunoregulation and often receive corticosteroids and other immunomodulatory therapies, which can deteriorate their immune responses, increasing their susceptibility to microbial infection. Furthermore, the incidence of RA in China is increasing and many patients with acute RA require hospitalization, which increases their opportunity for nosocomial infection. There are many tertiary hospitals providing services for people in Shanghai, the biggest city in China. However, there is no systemic investigation on the risk factors associated with the susceptibility of RA patients to nosocomial infection.

**Figure 1:**
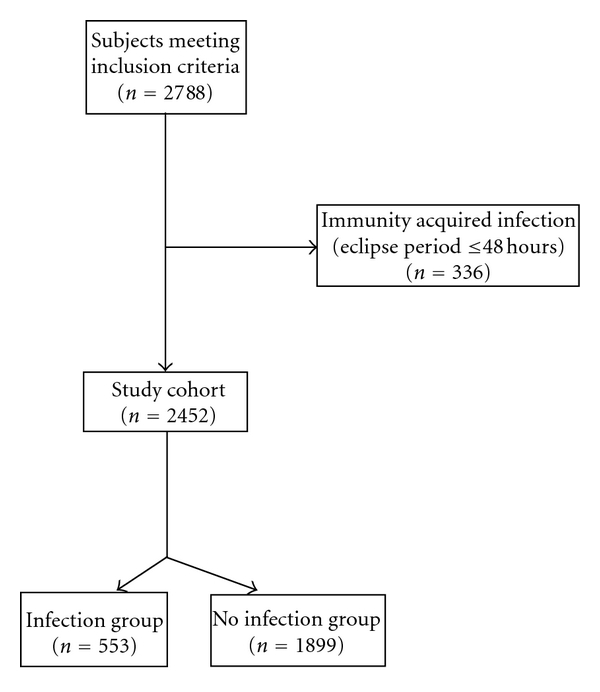
Flow diagram of study on RA patients at hospitals in Shanghai.

In this study, we aimed at analyzing the potential risk factors associated with nosocomial infection in patients with active RA (Figure 1).

## 2. Subjects and Methods

We analyzed the medical charts of 2452 patients who had been diagnosed with RA, according to the criteria of the American College of Rheumatology [6] at hospitals in Shanghai between January 2009 and February 2011. Individual patients were included if they had RA for more than three months with a disease activity score >3.2, based on erythrocyte sedimentation rate and an evaluation of 28 joints (DAS28) [7]. Individuals with other diseases were excluded. Written informed consent was obtained from individual patients, and the experimental protocol was approved by the Ethics Committee of the Second Military Medical University of Medicine, Shanghai, China.

Those patients with a history of nosocomial infection at 48 h or later after admission, according to the criteria of CDC definitions for nosocomial infections [8], were counted as patients with infection, while other patients who had no relevant infectious episode throughout the hospitalization were in the noninfection group. The criteria for an infectious episode were documentations of the microorganism and/or clinical findings in combination with either radiographic and endoscopic diagnosis or response to antibiotics. Opportunistic organisms were designated, according to Cohen's lists based on the principles proposed by von Graevenitz [9]. The type, site, and outcome of all nosocomial infections were recorded for each case.

Data are expressed as the real case, %, or mean ±SD of each group of patients. Categorical variables were analyzed using the *χ* ^2^ test and Fisher's exact test, where appropriate. Continuous variables were analyzed using Student's *t*-test. A 2-tail *P* value of 0.05 was considered to be statistically significant. The demographic and clinical characteristics of patients were taken into account as the potential risk factors, and they included age, gender, the duration of diseases, number of comorbidities, the duration of hospitalization, number of organs involved, number of disease modifying antirheumatic drugs (DMARDs), corticosteroid therapy, pulse cyclophosphamide therapy, Etanercept therapy, the peripheral white blood cell counts (WBCs), platelet, and eosinophils counts, the concentrations of serum globin, hemoglobin, serum albumin, C-reactive protein (CRP), erythrocyte sedimentation rate (ESR), immunoglobulin (Ig) G, IgA, and IgM. We first determined the potential risk factors associated with infection using a univariate analysis and further analyzed the association of these potential risk factors identified with infection in this population by a multivariate analysis using stepwise logistic regression. The statistical significance of risk factors associated with infection was assessed by means of 95% confidence intervals.

## 3. Results

The predisposition of female patients, the duration in hospital, the number of organs affected and DMARDs, the dosage of corticosteroids, the frequency of cases with pulse cyclophosphamide, the blood WBC and eosinophil counts, the concentrations of HB, ALB, CRP, and ESR in the patients with nosocomial infection were significantly greater than that in noninfection patients, determined by univariate analysis (Table 1). However, there was no significant difference in the mean age, the duration of the disease, the number of comorbidities, Etanercept therapy, and the level of blood PLT counts, globin, IgG, IgA, and IgM between the RA patients with noninfection and with infection. A multivariate logistic regression analysis revealed that the predisposition of female patients, the duration in the hospital, the number of organs affected, the number of DMARDs, corticosteroid therapy, blood WBC counts, and the concentrations of blood ALB and CRP were significantly independently associated with increased risk for infection in this population.

## 4. Discussion

A previous study has shown that RA patients have a nearly 2-fold increased risk for infection, compared with that of age- and sex-matched control subjects [10]. The increased risk for infections may be due to intrinsic and extrinsic defects. First, many RA patients show an accelerated immunosenescence, particularly in T cells [11]. These abnormalities may explain the high susceptibility of RA patients to nosocomial infection before treatment with immunosuppressive therapeutics. Second, aging, pulmonary disease, and diabetes mellitus are independent risk factors for bacterial infection in RA patients [12]. In our study, we found that female patients, longer duration in hospital, more organs affected by RA, more number of DMARDs, with corticosteroid therapy, and higher blood WBC counts, the higher levels of ALB and CRP were associated significantly with increased risk for the development of nosocomial infection. Indeed, male patients have less risk for nosocomial infection than women (OR = 0.70, 95% CI 0.53–0.92), and individual patients hospitalized for more than 12.31 (SD, 6.32) days have an increased risk for nosocomial infection (OR = 1.03, 95%CI 1.01–1.05). Furthermore, patients with less organs affected by RA were at a decreased risk for nosocomial infection (OR = 0.82, 95%CI 0.72–0.92). Moreover, we found that the RA patients with infection were significantly associated with the use of DMARD (OR = 1.22, 95%CI 1.061–1.40) and the dose of corticosteroids (OR = 1.02, 95%CI 1.01–1.03), which are consistent with these observations [13, 14], but in disagreement with another study [15]. Díaz-Lagares et al. observed an increased risk for infection in hospitalized RA patients, which was only associated with a slightly increased risk for the Etanercept therapies (HR 1.54, 95% CI 1.20–1.97) [16]. However, we observed no association of the Etanercept therapy with the increased risk for infection in RA patients. We also observed that serum hypoalbumin (OR = 0.98, 95%CI 0.97–0.99), high counts of WBC (especially, neutrophils, OR = 1.04, 95%CI 1.00–1.08), and high levels of serum CRP (OR = 1.03, 95%CI 1.01–1.04) were associated with increased risk for infection in RA patients. Hence, the abnormal levels of these measures may be used for the evaluation of infection in RA patients.

 All previous reports of infection are studied in RA patients in out-patient clinic (OPD), secondary, tertiary referral centers, or community. These studies may not provide a reliable estimation of the frequency of infection in RA patients. In the present study, we analyzed risk factors in hospitalized RA patients. This should allow us to predict the infection with other rheumatic disease patients.

We recognized that the present study had limitations. First, we conducted this observational study, based on a observational chart review data so that these findings could still be affected by unmeasured or unknown confounders, although we adjusted for all known risk factors for infection available in our administrative data. Furthermore, the analysis did not control for some disease characteristics at baseline specifically. We described disease activity or severity and the exposure to DMARDs as the number of organs affected and number of DMARDs, respectively. Finally, because the majority of residents in Shanghai are Chinese Han, our findings may be not generalized to the whole Chinese population.

## 5. Conclusion

Our results indicated female patients, longer duration in hospital, more organs affected by RA, more DMARDs and corticosteroid therapy, high counts of WBC, and lower concentrations of blood ALB, and higher levels of serum CRP were significantly and independently associated with increased risk for infection in RA patients. Apparently, these abnormal measures may be used for the diagnosis of infection in active RA patients. Given that infection is a serious challenge for the management of RA patients, it is important to identify infection and determine the susceptibility of pathogens to antibiotics for the control of infection in RA patients. Of course, further longitudinal prospective studies with diverse and big populations to confirm our findings are warranted in order to monitor and to prevent nosocomial infection in RA patients in China.

## Figures and Tables

**Table 1:** Demographic, clinical characteristics and risk factors for nosocomial infection in RA patients.

Variable	Total patients	Infection	No infection	Univariate analysis	Multivariate logistic regression analysis	
	(*N* = 2452)	(*n* = 553)	(*n* = 1899)	*P* value	Odds ratio (95%CI)	*P* value
Ages, mean (SD), years	59.81 (12.71)	59.66 (13.51)	58.69 (12.47)	0.113		
Female, %	2034 (82.90)	476 (86.07)	1558 (82.04)	0.026	0.70 (0.53–0.92)	0.012
Course of diseases, median (IQR), years	9.00 (3.00–16.00)	9.00 (3.00–16.00)	9.00 (3.00–17.00)	0.344		
Duration in hospital, mean (SD), days	11.49 (5.27)	12.31 (6.32)	11.25 (4.89)	<.001	1.03 (1.01–1.05)	<.001
Organs involved, No. mean (SD)	3.83 (0.67)	4.75 (0.79)	3.85 (0.63)	0.006	0.82 (0.72–0.93)	0.003
Comorbidities, No.mean (SD)	0.56 (0.81)	0.60 (0.81)	0.54 (0.80)	0.143		
DMARDs, No. mean (SD)	1.90 (0.721)	2.00 (0.743)	1.87 (0.71)	<.001	1.22 (1.06–1.40)	0.005
Corticosteroids dosage, mean (SD), mg/d	3.55 (8.43)	4.33 (9.97)	3.31 (7.92)	0.027	1.02 (1.01–1.03)	0.003
Pulse CTX, %	95 (3.87)	31 (5.6)	64 (3.3)	0.017		
Etanercept, %	1226 (50.00)	263 (47.56)	963 (50.71)	0.192		
WBC counts, median (IQR), ×10^9^/L	6.40 (5.00–7.90)	6.60 (2.51–8.20)	6.38 (3.00–7.80)	0.005	1.04 (1.00–1.08)	0.051
PLT, median (IQR), ×10^12^/L	232.00 (173.00–302.00)	236.00 (171.00–306.00)	228.00 (174.00–302.00)	0.557		
E., median (IQR), ×10^9^/L	0.14 (0.09–0.25)	0.14 (0.09–0.29)	0.14 (0.09–0.25)	0.013		
HB, median (IQR), g/L	113.00 (102.00–125.00)	110.00 (100.00–122.00)	114.00 (103.00–126.00)	<.001		
Globin, median (IQR), g/L	30.00 (27.00–34.50)	30.00 (26.00–34.00)	30.10 (27.00–34.60)	0.088		
ALB, median (IQR), g/L	35.00 (30.00–39.20)	34.00 (28.00–38.00)	35.80 (31.00–40.00)	<.001	0.98 (0.97–0.99)	<.001
ESR, median (IQR), mm/H	36.00 (15.00–67.00)	38.00 (17.20–76.00)	36.00 (14.00–65.00)	0.006		
CRP, median (IQR), mg/L	8.00 (2.53–28.10)	12.10 (.35–41.00)	7.88 (2.28–25.3)	<.001	1.03 (1.02–1.04)	0.021
IgG, median (IQR), mg/L	12.60 (4.76–16.00)	12.10 (6.80–15.70)	12.70 (2.80–16.00)	0.873		
IgA, median (IQR), mg/L	2.68 (0.87–3.70)	2.74 (1.01–3.81)	2.60 (0.7–3.70)	0.356		
IgM, median (IQR), mg/L	1.00 (0.20–1.64)	1.00 (0.40–1.56)	1.04 (0–1.67)	0.532		

Data are number (%) of patients, unless otherwise indicated. SD: standard deviation. IQR: interquartile range. CI: confidence interval. Organs involved, No.: the number of involved organs. Comorbidities, No.: the number of comorbidities: hypertension, diabetes mellitus, malignancy, chronic lung disease, congestive heart failure, cardiac arrhythmia, renal failure, liver disease. DMARDs, No.: the numbers of DMARs (disease-modifying antirheumatic drugs). WBC: white blood cell. PLT: platelet. E: eosinophils. HB: homoalbumin. ALB: albumin. ESR: erythrocyte sedimentation rate. CRP: C-reactive protein.
